# Human Infertility and Reproductive Endocrinology

**DOI:** 10.3390/life14121550

**Published:** 2024-11-26

**Authors:** Giuseppe Grande

**Affiliations:** Unit of Andrology and Reproductive Medicine, Department of Medicine, University of Padua, Via Giustiniani 2, 35128 Padua, Italy; giuseppe.grande@aopd.veneto.it

## 1. Introduction

One of the biggest problems facing modern medicine is infertility. It is defined by the lack of conception after at least 12 months of regular, unprotected sexual intercourse [1]. About 10–15% of couples worldwide are infertile, with 56% of them seeking medical care [2]. The World Health Organization (WHO) has declared infertility to be a disease entity and a social illness because of its high frequency. Since starting a family and having children is a basic human necessity, it is covered under the Universal Declaration of Human Rights.

Furthermore, because it always affects two persons at the same time—a woman and a man—it is regarded as a particular and distinct health issue. In addition, data reported that infertile patients have a significantly higher incidence of comorbidities than fertile subjects [3]. As such, the clinical care of an infertile couple should not only be focused on their reproductive function but should also include health promotion strategies and general health management.

Reproductive endocrinology is one of the original officially recognized subspecialties in the areas of both endocrinology and gynecology. Originally focused on endocrine problems related to reproductive function, assisted reproductive technologies (ART) have recently become a larger part of training during reproductive endocrinology training fellowships [4]. Since the introduction and development of ART, in fact, several attempts have been made to apply the best technical procedures to improve the success of the techniques in terms of pregnancy rate and “baby in arm” per cycle rate [5].

Furthermore, the spread of ART over the last 40 years showed several limits of these techniques. Over the past two decades, in fact, there have been several concerns about the overuse of ART [6] because of the lack of evidence regarding effectiveness in certain populations (e.g., unexplained infertility [7]), potential short- and long-term safety [8], and economic considerations [9]. Furthermore, for some patients, ART might raise ethical problems that must be considered to safeguard the patient–doctor relationship and to respect the patient’s autonomy to make mature and responsible choices [10].

Moreover, previous data showed that up to 27% of male partners of couples seeking fertility care do not receive an andrological evaluation [11]. Evaluation of the male, in fact, is often neglected because women are considered the only “patients” in fertility treatment. Additionally, the public and provider perception of infertility is that it is primarily a gynecological problem. Therefore, infertility is not a matter for the woman or the man alone; it is a matter for the couple, so the clinical approach should be personalized to consider different aspects specific to each couple.

Starting from these premises, this Special Issue has captured the diversity of the studies that focus on human fertility and infertility. It contains four articles and two reviews, which I briefly describe in the next few paragraphs, mainly focused on improvements in ART and in the physiology of female fertility.

## 2. An Overview of Published Articles

Anastasia Sysoeva’s article (contribution 1) investigated the molecular composition of follicular fluid (FF) extracellular vesicles (EVs) in women of different reproductive ages and its possible relationship to sperm fertilizing ability. The researchers performed a lipidomic and proteomic analysis of FF EVs. They reported that, in women younger than 35 years, the concentration of vesicular progesterone was 6.6 times higher than in women older than 35 years, and the total levels of the main lipid classes were increased in younger women. Furthermore, the authors studied the changes in proteins involved in sperm activation, shedding light on the possible interaction between the FF EVs of women in different age groups and male germ cells.

Michela Palese et al., in their article (contribution 2), compared the semaphoring 3A (SEMA3A) plasma levels in 77 infertile women with diminished ovarian reserve (DOR) and in healthy volunteers, revealing that the SEMA3A levels were significantly higher in patients with DOR. Furthermore, the SEMA3A levels were increased in patients who underwent fertility treatment and had positive Beta-Human Chorionic Gonadotropin (βHCG) values (β+) after controlled ovarian stimulation (COS) compared to those who had negative βHCG values (β−). These data might open interesting perspectives for future investigations, both in understanding the physiological role of SEMA3A in female reproduction and in confirming its role as a putative marker for DOI and response to fertility treatments.

The third article is from Martin Stimpfel and aims to understand the best day to perform embryo transfer (ET) during the cycles of ART, when there are only one or two embryos obtained in the cycle. The authors retrospectively evaluated the stimulated IVF/ICSI cycles performed in their institution in the period between 1 January 2004 and 31 December 2018, in which one or two embryos were obtained, and compared the data between day three and day five embryo transfer (ET). They reported that the birth rate per ET was significantly higher in the day five ET group, and further analysis indicated that this could be due to the trend observed in a group of patients under 36 years old, while in older patients there was no such difference. As a consequence, this study suggests that there is an advantage in performing ET on day five versus day three, even when 1–2 embryos are produced, but probably only for younger patients under 36 years old.

The fourth text published in this Special Issue is original research by Yolanda Fernandez-Hermida aimed at characterizing the biochemical changes in cervical mucus (CM) composition along the cycle. The authors studied CM samples collected from the lumen of the cervical canal from women of reproductive age on three different days of the same menstrual cycle. Samples were first analyzed and classified by light microscopy. High-resolution mass spectrometry and bioinformatic analysis were performed afterwards to determine the in vivo changes in CM protein composition. Under a light microscope, the authors reported the specific cyclical changes in CM in its crystallographic patterns and correlated these changes with the differences reported, at a biochemical level, by proteomic analysis. In detail, they reported that twenty-five CM proteins identified are potential biomarkers of ovulation, thus opening interesting perspectives both in the field of fertility awareness and in the field of infertility pathophysiology.

The article by Romualdo Sciorio et al. is a review providing an analytical revision of the current literature relating to the correlation between ovarian stimulation procedures during ART and oocyte/embryo quality. They analyzed different aspects of oocyte quality, including morphological features, oocyte competence, and its surrounding environment. Furthermore, they studied the main noninvasive features as well as novel approaches to biomechanical parameters of oocytes that might be correlated with the competence of embryos to produce a healthy pregnancy and live birth.

Finally, the article by Antonio D’Amato is a review about fertility preservation (FP) in transgender men who desire biological offspring in the future. Although the frequency of transgender individuals in the population is increasing, there is currently no personalized approach to FP for transgender men, and the available techniques have limitations that require further investigation. In their review, the authors highlight the shortcomings of current methods and the areas where additional research is needed to advance the field, finally focusing on the importance of providing patients with accurate information about the benefits and potential risks of different FP techniques, understanding and considering the patient’s reproductive goals, from the perspective of personalized medicine.

## 3. Conclusions

This compilation of articles encompasses a diverse range of research, elucidative of the richness of the research field. This is also reflected in the different methodologies that were adopted for the studies, ranging from clinical studies to molecular studies by proteomic and lipidomic techniques.

The collection of articles proposed for this Special Issue reflects moreover the complexity of the present topic, including the study of female and male reproductive physiology, the molecular basis of male and female infertility, and the clinical relevance of the different clinical conditions causing infertility, the identification of novel putative markers for the different clinical conditions associated with infertility [12], the diagnostic and therapeutic management of the infertile couple [13], including both medical and surgical treatments and ART, the possibilities to improve ART success, and the preservation of fertility for patients undergoing potentially gonadotoxic treatments [14].

Thus, these aspects highlight the relevance and importance of this Special Issue, allowing the reader to find studies covering different aspects in the field of fertility and infertility, which allows a more complete view on the research field of reproductive endocrinology.

Further research should be focused more on understanding the pathophysiology of infertility, especially for male factor infertility, and in possible medical and surgical treatments aimed at the restoration of natural fertility, as an alternative to the overuse of ART in the last 40 years. These aspects might be the aim of a further thematic Special Issue.
